# A London Congress of Public Health

**Published:** 1905-06-17

**Authors:** 


### The Hospital.

A Journal of

The Medical Sciences and Hospital Administration.

Vol. XXXVIII.—No. 977. SATURDAY, JUNE 17, 1905.

### A London Congress of Public Health.

The Royal Institute of Public Health, of which Dr. William R. Smith, F.R.S.Edin., appears to be permanent "President," and which has its headquarters at 37 Russell Square, has issued what at first sight appears to be an exceedingly ambitious programme of a projected Sanitary Congress, to be held in London from July 19th to the 25th. The Institute itself is perhaps not so widely known as it deserves to be, or as it certainly will be if the projected Congress should fulfil the hopes of the projectors; but it has the advantage of support from Royalty, His Majesty the King being Patron, and the Prince of Wales Vice-Patron. The Marquis of Londonderry has accepted the position of President of the Congress, and has consented to deliver an inaugural address at the first meeting, which, by the permission of Mr. Beerbohm Tree, will be held in His Majesty's Theatre, Haymarket, at 3 p.m. on July 19th. The list of Vice-Presidents of the Congress includes 138 very distinguished people, commencing with archbishops and dukes, comprising many other persons of social or scientific eminence, and concluding with the Worshipful Mayors of Metropolitan Boroughs and the Masters of three City Companies, although the names of acknowledged sanitary authorities, such as Medical Officers of Health, are chiefly conspicuous by their absence. There is a Metropolitan Mayors' Reception Committee, of which the Lord Mayor of London is Chairman, and the Mayor of the City of Westminister (Major-General Lord Cheylesmore) is Vice-Chairman. Mr. James Cantlie, M.A., M.B., has undertaken the arduous office of honorary secretary, and the scientific work of the Congress is to be divided into eight sections, distinguished, like those of the British Association for the Advancement of Science, by the earlier letters of the alphabet. Section A (Preventive Medicine), will be presided over by Sir James Crichton Browne; Section B (Municipal Administration of the Education Acts), by Mr. Yoxall, M.P.; Section C (Child Study and School Hygiene), by Lord Stanley of Alderley; Section D (Engineering and Building Construction), by Sir Alexander Binnie; Section E (Bacteriology and Chemistry), by Professor Hewlett; Section F (Veterinary Hygiene), by Professor McFadyean; Section G (Tropical Medicine), by Col. Crombie, M.D.; and Section H (Naval and Military Hygiene), by Lieut.-Col. Firth, F.R.C.S. In addition to the fare which these sections will provide for scientific workers, numerous festivities have been arranged.

It is trite to observe that nothing succeeds like success, and if the framers of this programme can carry it out in its entirety they will not only deserve great credit, but will be likely actually to accomplish something in the way of bringing home to Londoners the vast importance of sanitary science and the magnitude of the issues with which it deals. There can be no doubt that the provincial meetings of the British Association have a very real educational influence in the various local centres in which they are held and in which, for the time, they are the great events of the days on which they occur. They hold the ground and there is nothing to compete with the attractions which they offer. But London is the centre of too many interests to permit of its attention being engrossed by any of them, and it is only too probable that the great bulk of the population will proceed upon their respective ways and be engrossed in their respective duties or pleasures without so much as recognising the fact that a Congress of Public Health is being held in the midst of them, and that, by a moderate payment, and by resorting to King's College or to the Polytechnic Institution, they may make themselves acquainted with the very latest views or discoveries in the various subjects with which the sections propose to deal. Much will depend, of course, upon the amount of importance which the non-medical Press may see fit to attach to the proceedings, and upon the degree of accuracy and of fulness with which they will be reported. With regard to this aspect of the question, we are inclined to fear that the programme covers too much ground, and we would respectfully suggest to our contemporaries that much room for judicious selection will be afforded to them. In each section, we are told, special discussions will be arranged, in addition to any papers which may be volunteered; and it may be presumed that the speakers who are invited to conduct the discussions will be carefully selected, and will be representative of advanced knowledge on the subjects with which they will be called upon to deal. If this expectation be fulfilled, it is obvious that the three first sections will be those most worthy of public attention; not, perhaps, on the mere ground of their importance, but because they fall more than the others within the cognisance of the average citizen, and within the scope of his ordinary activities. In other words, they deal with matters which are less technical than the construction of buildings or the ventilation of ships. The prevention of disease would be enormously promoted if every householder would reform his own household with regard to it; and the health of the rising generation would be promoted in an equal degree if every parent would consider it as an interest appertaining to himself, and therefore not to be absolutely delegated to County Councillors and to schoolmasters. This is the lesson which the Congress may most fitly teach, and with regard to which we hope that a large measure of success may be attendant upon its efforts.

